# On the development of a semi-nonparametric generalized multinomial logit model for travel-related choices

**DOI:** 10.1371/journal.pone.0186689

**Published:** 2017-10-26

**Authors:** Ke Wang, Xin Ye, Ram M. Pendyala, Yajie Zou

**Affiliations:** 1 Key Laboratory of Road and Traffic Engineering of Ministry of Education, College of Transportation Engineering, Tongji University, Shanghai, China; 2 School of Sustainable Engineering and the Built Environment, Arizona State University, Tempe, Arizona, United States of America; Beihang University, CHINA

## Abstract

A semi-nonparametric generalized multinomial logit model, formulated using orthonormal Legendre polynomials to extend the standard Gumbel distribution, is presented in this paper. The resulting semi-nonparametric function can represent a probability density function for a large family of multimodal distributions. The model has a closed-form log-likelihood function that facilitates model estimation. The proposed method is applied to model commute mode choice among four alternatives (auto, transit, bicycle and walk) using travel behavior data from Argau, Switzerland. Comparisons between the multinomial logit model and the proposed semi-nonparametric model show that violations of the standard Gumbel distribution assumption lead to considerable inconsistency in parameter estimates and model inferences.

## 1. Introduction

The Gumbel distribution (also referred to as the Type-I extreme value distribution) plays a central role in discrete choice models for travel demand analysis[1]. This can be attributed to two major reasons. First, the Gumbel distribution closely resembles the normal distribution, which is often the preferred distribution to characterize the random disturbance term in an econometric model that accounts for the effect of unobserved factors. Second, when the Gumbel distribution is assumed for random components of utility functions, a closed-form likelihood function is obtained in the context of the application of the microeconomic utility maximization principle. With a closed-form likelihood function, maximum likelihood estimation (MLE) methods can be applied with ease to estimate model coefficients consistently and efficiently. Due to these appealing features of the Gumbel distribution, the Multinomial Logit (MNL) model is widely applied in practice and preferred over its counterpart that is based on the assumption of a normally distributed random error component (i.e., Multinomial Probit or MNP model)[2–4]. In the context of discrete-continuous choice behaviors, the Multiple Discrete-Continuous Extreme Value (MDCEV) model[5–9] developed based on the standard Gumbel distribution has a neat closed-form log-likelihood expression while others based on the normal distribution assumption do not have this feature[10–17].

However, according to the theory of maximum likelihood estimation, the consistency and efficiency of maximum likelihood estimators depend on the validity of the distributional assumption made on the random error term. It is important to ensure that the distributional assumptions on the random error terms are valid when applying the MLE method to estimate model coefficients of a discrete choice model. Methods to test for violations of the normal distribution are currently available in the economic literature[18]. Recently, the authors developed a practical method to test the validity of the distributional assumption on the random disturbance term in an MNL model and obtained significant statistical evidence to reject the standard Gumbel distribution assumption in a very commonly encountered empirical setting dealing with long distance travel mode choice[19]. That finding motivates this particular study which aims to develop and present the formulation for a Semi-nonparametric Generalized Multinomial Logit Model (SGMNL) for travel-related choices. The objective of this study is to generalize the MNL model by relaxing the assumption of a Gumbel distribution using a semi-nonparametric approach, and then demonstrate the efficacy of the approach by applying the generalized model to an empirical setting of travel mode choice. It should be noted that this generalization essentially differs from other extensions of the MNL that have yielded the Nested Logit, Cross-nested Logit, Heteroskedastic Logit or Multinomial Probit models[20]. Those models are generalized extensions that persistently employ the unimodal Gumbel or normal marginal distributions, whereas the proposed semi-nonparametric model presented in this paper allows the marginal error distribution to have multiple modes. Thus, the proposed model provides the ability to examine potential bias in model coefficients, marginal effects and elasticities in a discrete choice model that may arise when a unimodal distribution like the standard Gumbel distribution is violated in random components of utility functions.

Discrete choice models are widely used in transportation planning practice to predict travel mode choice behavior; the choice of transport mode has important implications for traffic congestion, energy consumption and air pollution. The study of mode choice behavior and its determinants can help transportation planning professionals design alternatives and implement policies that enhance sustainability, livability, and public health while reducing delays due to congestion. There are a number of recent studies in the literature that have focused on a study of travel mode choice behavior. For example, Shen et al. (2016) found that proximity to metro stations has a significant positive effect on the choice of rail transit as a primary commuting mode[4]. Ding et al. (2017) applied an integrated structural equation model and discrete choice model to investigate how the built environment affects travel mode. In their model system, they account for the mediating effects of car ownership and travel distance, thereby capturing both the direct and indirect effects of built environment attributes on travel mode choice[2]. Ding et al. (2014) proposed a cross-classified multilevel probit model of travel mode choice[21]. Comparisons with a traditional mode choice model not only revealed the effects of residential and workplace location on tour-based commute mode choice behavior, but also revealed the presence of spatial heterogeneity across home location and workplace in mode choice behavior. In this paper, a semi-nonparametric choice modeling method is proposed and applied to model commute mode choice among four alternatives (auto, transit, bicycle and walk) using data from Argau, Switzerland. The proposed approach is motivated by the desire to offer a more flexible and robust methodological framework for activity-travel behavior analysis.

The remainder of the paper is organized as follows. In Section 2, the literature on semi-nonparametric choice models is reviewed. In Section 3, the orthonormal Legendre polynomial is introduced and then applied to extend the standard Gumbel distribution, thus enabling the development and formulation of the Semi-nonparametric Generalized Multinomial Logit Model (SGMNL). In Section 4, data used for the empirical study is described, and empirical estimation results are presented and discussed. Finally, conclusions and directions for future research are presented in the last section.

## 2. Literature review

As early as the time when McFadden initially proposed the MNL model[22], econometricians have been questioning the validity of the distributional assumption on the error term in random utility functions[23]. When a violation of the standard Gumbel distribution assumption is found, alternative modelling approaches may be explored to overcome the ill-effects. Adopting an alternative parametric distribution for random utilities may prove to be a solution; for example, the Weibull or logistic distribution recently proposed in the literature[24, 25] could serve as appropriate distributional assumptions on the random error term. In addition, a generalized multinomial logit model or a discrete-continuous choice model that allows heteroscedastic variance may also prove to be superior to the standard MNL and MDCEV model[26, 27]. However, all of these alternative distributions are unimodal in nature and therefore cannot capture potential multimodalities in random errors.

Concerns about the adverse effects of violations of distributional assumptions on the random error components have motivated the development of semi-parametric and semi-nonparametric choice models. The semi-parametric choice model employs the kernel density method to estimate the distribution of random errors, and therefore does not rely on any parametric distributional assumptions[28–32]. The semi-nonparametric (SNP) choice model, on the other hand, is developed based on a polynomial approximation of a probability density function (PDF) that takes a flexible form[33]. Because the likelihood function has an explicit analytical expression, the SNP choice modeling method appears to be more widely applied in practice than the semi-parametric approach[34–37].

Similar to a binary probit model, the SNP binary choice model formulation also starts with a random utility (U), which can be expressed as U = V + ε, where "V" is the systematic component and "ε" is the random component. If a dummy variable "y" indicates whether an alternative is chosen or not, then P(y = 1) = P(U > 0) = P(V + ε > 0) = P(ε > −V). The probability density function of "ε" takes the following form:

$$f(\varepsilon )=\frac{{\left(\sum_{i=0}^{K}a_{i}\varepsilon^{i}\right)}^{2}\varphi (\varepsilon )}{\int_{-\infty }^{+\infty }{\left(\sum_{i=0}^{K}a_{i}\varepsilon^{i}\right)}^{2}\varphi (\varepsilon )d\varepsilon }.$$ (1)

In Eq (1), φ(ε) represents the PDF of the standard normal distribution and is referred to as the "a priori distribution". The denominator ensures that $\int_{-\infty }^{+\infty }f(\varepsilon )d\varepsilon =1$. Eq (1) can be extended as follows:

$$f(\varepsilon )=\frac{\left(\sum_{i=0}^{K}\sum_{j=0}^{K}a_{i}a_{j}\varepsilon^{i+j})\varphi (\varepsilon \right)}{\int_{-\infty }^{+\infty }(\sum_{i=0}^{K}\sum_{j=0}^{K}a_{i}a_{j}\varepsilon^{i+j})\varphi (\varepsilon )d\varepsilon }.$$ (2)

$$Then,\;P(y=1)=P(\varepsilon >-V)=\frac{\int_{-V}^{+\infty }\left(\sum_{i=0}^{K}\sum_{j=0}^{K}a_{i}a_{j}\varepsilon^{i+j})\varphi (\varepsilon \right)d\varepsilon }{\int_{-\infty }^{+\infty }(\sum_{i=0}^{K}\sum_{j=0}^{K}a_{i}a_{j}\varepsilon^{i+j})\varphi (\varepsilon )d\varepsilon }=\frac{\sum_{i=0}^{K}\sum_{j=0}^{K}a_{i}a_{j}\int_{-V}^{+\infty }\varepsilon^{i+j}\varphi \left(\varepsilon \right)d\varepsilon }{\sum_{i=0}^{K}\sum_{j=0}^{K}a_{i}a_{j}\int_{-\infty }^{+\infty }\varepsilon^{i+j}\varphi \left(\varepsilon \right)d\varepsilon }.$$ (3)

To evaluate the probability value above, recursion formulas may be applied to derive the indefinite integral of ∫ ε^i+j^φ(ε)dε. The above SNP choice model is limited to a binary choice situation due to its computational complexity in the context of a multinomial choice situation.

## 3. Modeling methodology

### 3.1 Extending the standard gumbel distribution with the orthonormal legendre polynomial

Bierens[38] proposed a new polynomial, called the orthonormal Legendre polynomial, for estimating distributions on the unit interval in a semi-nonparametric framework. In the transportation choice modeling literature, this approach has been used to test normal and log-normal distributions of random coefficients in mixed logit models[39]. As per Fosgerau and Bierlaire[39] and Bierens[38], the orthonormal Legendre polynomial may be recursively defined as:

$$L_{0}=1,\;L_{1}=\sqrt{3}(2x-1),$$ (4)

$$L_{n}=\alpha_{n}(2x-1)L_{n-1}+\beta_{n}L_{n-2},\;n\geq 2$$ (5)

In Eq (5), $\alpha_{n}=\frac{\sqrt{4n^{2}-1}}{n}$, $\beta_{n}=-\frac{(n-1)\sqrt{2n+1}}{n\sqrt{2n-3}}$. The advantage of using this polynomial is that it ensures

$$\int_{0}^{1}L_{m}(x)L_{n}(x)dx=\left\{ \begin{array}{c} 0\;\mathrm{if}\;m\neq n\\ 1\;\mathrm{if}\;m=n \end{array}\right..$$ (6)

According to Gallant and Nychka[33], the prior distribution in the semi-nonparametric approach can be a distribution other than the standard normal distribution. In this paper, the orthonormal Legendre polynomial is used to construct a semi-nonparametric (SNP) probability density function that extends the standard Gumbel distribution as follows:

$$f(x)=\frac{{\left\{1+\sum_{k=1}^{K}\delta_{k}L_{k}[G(x)]\right\}}^{2}}{1+\sum_{k=1}^{K}{\delta_{k}}^{2}}g(x),$$ (7)

where g(x) = exp(−e^−x^) ∙ exp(−x), G(x) = exp(−e^−x^), δ_k_ are scalar parameters and K represents the total number of polynomials. Using Eq (6), it can be shown that $\int_{-\infty }^{+\infty }f(x)=1$. As f(x) is positive, it qualifies as a probability density function.

**Fig 1:**
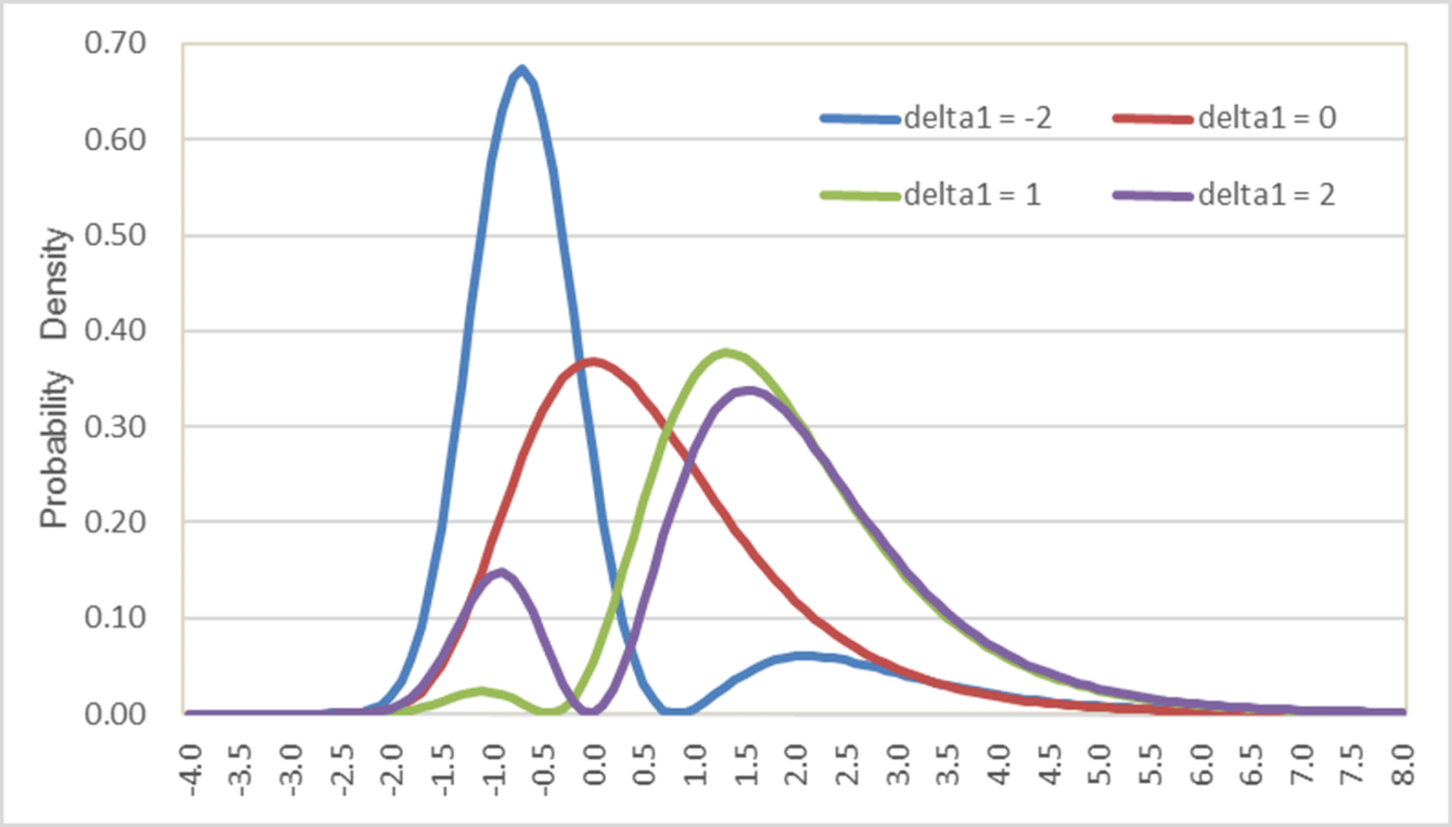
Comparisons of semi-nonparametric probability densities when K = 1.

Fig 1 compares the semi-nonparametric probability densities when the number of polynomials is 1 (K = 1) and the parameter δ_1_ takes a value of -2, 0, 1 or 2. When δ_1_ is 0, the distribution reduces to a standard Gumbel distribution, as shown by the red curve. When δ_1_ takes a value of -2, 1 or 2, the distributions are bimodal, although the secondary peak in the distribution is rather flat when δ_1_ is equal to -2 or 1.

**Fig 2:**
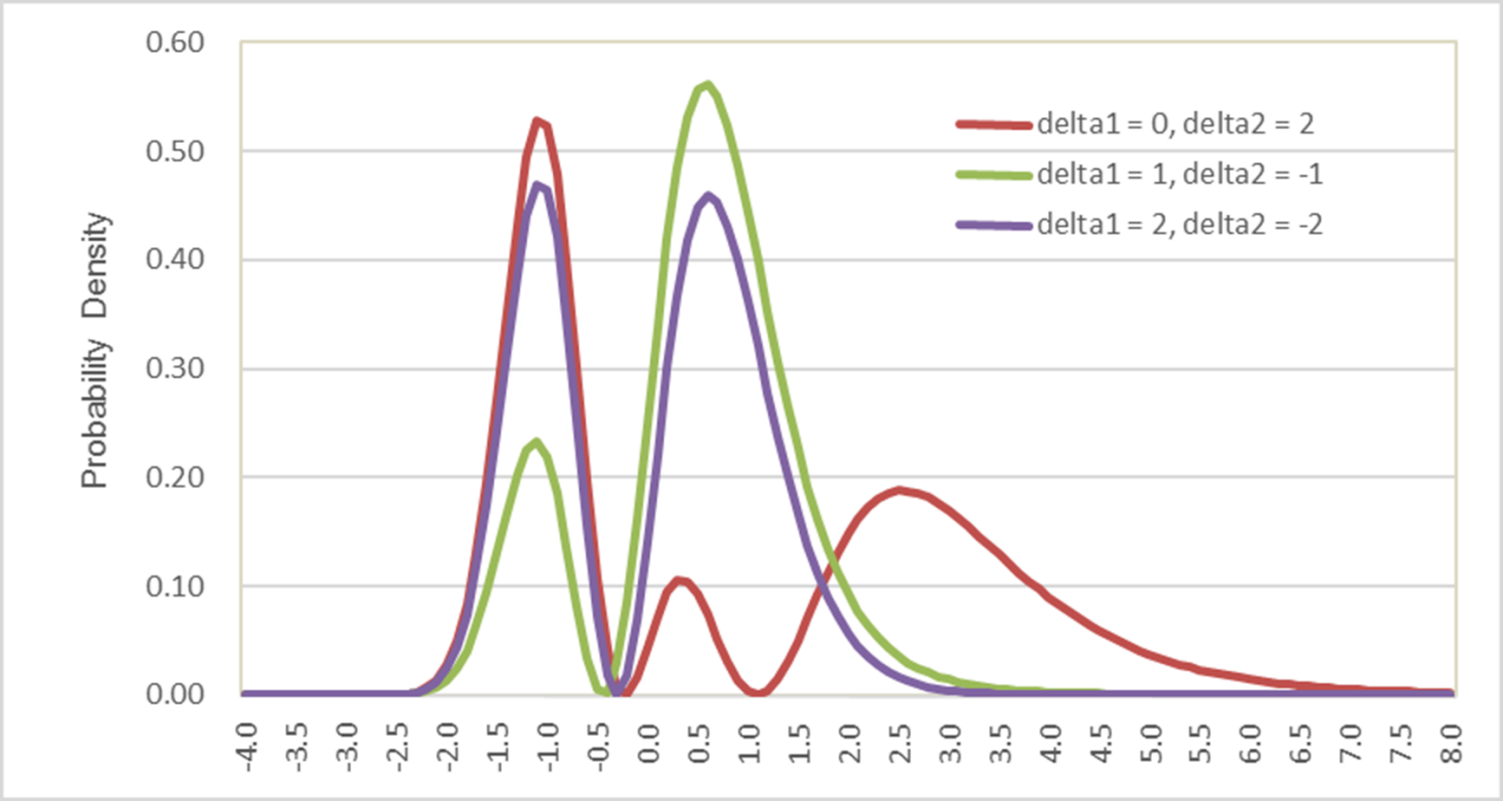
Comparisons of semi-nonparametric probability densities when K = 2.

Fig 2 compares the semi-nonparametric probability densities when the number of polynomials is 2 (K = 2) and two scalar parameters δ_1_ and δ_2_ are involved. With two polynomials, and where the highest power term of “G(x)” increases to 2, the SNP function represented in Eq (7) can generate a more flexible probability density distribution. It can be seen that, when δ_1_ is 2 and δ_2_ is -2, the distribution exhibits two modes with almost equal probability densities. When δ_1_ is 0 and δ_2_ is 2, the distribution shows three modes. It may further be expected that, when the number of polynomials (K) or the highest power term of “G(x)” increases, the SNP function with a flexible form can effectively represent the probability density function for a large family of distributions with multiple modes. Such flexibility allows for a better representation of the distribution of the error term in a random utility function of a choice model, and therefore provides the ability to obtain more consistent estimates of model coefficients.

### 3.2 Simplifying the semi-nonparametric *(SNP)* probability density function *(PDF)*

Following Gallant and Nychka[33], it is possible to employ the SNP PDF in Eq (7) to construct random components in utility functions so that multiple modes may be accommodated in their distributions. Before the choice probability can be derived, the SNP PDF needs to be simplified first. Using Eqs (4) and (5), it is possible to write the polynomial in a general form as:

$$L_{n}=\sum_{k=0}^{n}c_{n,k}x^{k},$$ (8)

where c_n,k_ is a constant coefficient for the term “x^k^” in the n^th^ polynomial. When k > n, c_n,k_ = 0. Let $a=2\sqrt{3}$ and $b=-\sqrt{3}$. Then, L_0_ = 1 and L_1_ = *a*x + *b*. When n ≥ 2, as per Eq (5),

$$\begin{array}{l} L_{n}=\alpha_{n}(2x-1)L_{n-1}+\beta_{n}L_{n-2}=\alpha_{n}(2x-1)\sum_{k=0}^{n-1}c_{n-1,k}x^{k}+\beta_{n}\sum_{k=0}^{n-2}c_{n-2,k}x^{k}\\ \;={2\alpha }_{n}\sum_{k=0}^{n-1}c_{n-1,k}x^{k+1}{-\alpha }_{n}\sum_{k=0}^{n-1}c_{n-1,k}x^{k}+\beta_{n}\sum_{k=0}^{n-2}c_{n-2,k}x^{k}. \end{array}$$

Since c_n−2,n−1_ = 0, $L_{n}={2\alpha }_{n}\sum_{k=0}^{n-1}c_{n-1,k}x^{k+1}{-\alpha }_{n}\sum_{k=0}^{n-1}c_{n-1,k}x^{k}+\beta_{n}\sum_{k=0}^{n-1}c_{n-2,k}x^{k}$ $=(-\alpha_{n}c_{n-1,0}+\beta_{n}c_{n-2,0})x^{0}+\sum_{k=1}^{n-1}[\alpha_{n}(2c_{n-1,k-1}-c_{n-1,k})+\beta_{n}c_{n-2,k}]x^{k}+2\alpha_{n}c_{n-1,n-1}x^{n}$.

Then, it is possible to write:

$$L_{n}(x)=c_{n,0}x^{0}+\sum_{k=1}^{n-1}c_{n,k}x^{k}+c_{n,n}x^{n}.$$ (9)

$$In\;the\;equation\;above,\;\left\{ \begin{array}{c} c_{n,0}=-\alpha_{n}c_{n-1,0}+\beta_{n}c_{n-2,0};\\ c_{n,k}=\alpha_{n}(2c_{n-1,k-1}-c_{n-1,k})+\beta_{n}c_{n-2,k}),0<k<n;\\ c_{n,n}=2\alpha_{n}c_{n-1,n-1}. \end{array}\right.$$ (10)

When n = 0 or 1, define c_0,0_ = 1, c_1,0_ = *b*, and c_1,1_ = *a*. For any integer “n” (n ≥ 2), the recursion equations (10) can be applied to compute the coefficients c_i,j_ and all of the c_i,j_ values form a lower triangular matrix, called the “c” matrix in this paper. Table 1 provides an example of such a “c” matrix when “n” reaches 6. With the “c” matrix, the general form of the orthonormal Legendre polynomial (given the “n” value) may be obtained. For example, when n = 4, the fourth row vector of coefficients in the “c” matrix can be extracted to write the polynomial as L_4_(x) = 3x^0^ − 60x^1^ + 270x^2^ − 420x^3^ + 210x^4^.

**Table 1:** An example of “c” matrix.

k	0	1	2	3	4	5	6
n							
0	1.00	0.00	0.00	0.00	0.00	0.00	0.00
1	-1.73	3.46	0.00	0.00	0.00	0.00	0.00
2	2.24	-13.42	13.42	0.00	0.00	0.00	0.00
3	-2.65	31.75	-79.37	52.92	0.00	0.00	0.00
4	3.00	-60.00	270.00	-420.00	210.00	0.00	0.00
5	-3.32	99.50	-696.49	1857.31	-2089.47	835.79	0.00
6	3.61	-151.43	1514.33	-6057.33	11357.49	-9994.59	3331.53

After the “c” matrix is generated, δ_0_ needs to be defined as 1 and the numerator in the SNP probability density function in Eq (7) can be rewritten as:

$${\left\{1+\sum_{k=1}^{K}\delta_{k}L_{k}[G(x)]\right\}}^{2}={\left\{\sum_{k=0}^{K}\delta_{k}L_{k}[G(x)]\right\}}^{2}={\left[\sum_{k=0}^{K}\delta_{k}\sum_{i=0}^{K}c_{k,i}{G(x)}^{i}\right]}^{2}={\left[\sum_{i=0}^{K}\left(\sum_{k=0}^{K}{\delta_{k}c}_{k,i}\right)G{\left(x\right)}^{i}\right]}^{2}.$$

Define a “d” vector, where each element $d_{i}=\sum_{k=0}^{K}{\delta_{k}c}_{k,i}$. Since c_k,i_ = 0 when k < i,

$$d_{i}=\sum_{k=i}^{K}{\delta_{k}c}_{k,i}.$$ (11)

Thus, ${\left\{1+\sum_{k=1}^{K}\delta_{k}L_{k}[G(x)]\right\}}^{2}={\left[\sum_{i=0}^{K}d_{i}G{\left(x\right)}^{i}\right]}^{2}=\sum_{i=0}^{K}\sum_{j=0}^{K}{d_{i}d_{j}G(x)}^{i+j}$. The SNP probability density function in Eq (7) may then be rewritten as:

$$f(x)=\frac{{\left\{1+\sum_{k=1}^{K}\delta_{k}L_{k}[G(x)]\right\}}^{2}}{1+\sum_{k=1}^{K}{\delta_{k}}^{2}}∙g(x)=\sum_{i=0}^{K}\sum_{j=0}^{K}\left[(\frac{d_{i}d_{j}}{\sum_{k=0}^{K}{\delta_{k}}^{2}}){G(x)}^{i+j}\right]g(x)=\{\sum_{m=0}^{M}{\xi_{m}[G(x)]}^{m}\}g(x).$$

$$In\;the\;formula\;above,\;\;M=2K\;and\;\xi_{m}=\left\{ \begin{array}{c} \frac{\sum_{i=0}^{m}d_{i}d_{m-i}}{\sum_{k=0}^{K}{\delta_{k}}^{2}},\;if\;m\leq K;\\ \frac{\sum_{i=m-K}^{K}d_{i}d_{m-i}}{\sum_{k=0}^{K}{\delta_{k}}^{2}},\;if\;K<m\leq 2K. \end{array}\right.$$ (12)

Essentially, the SNP PDF in Eq (7) has been simplified to be:

$$f(x)=\{\sum_{m=0}^{M}{\xi_{m}[G(x)]}^{m}\}g(x),$$ (13)

where ξ_m_ is a function with respect to parameters δ_k_, and M (= 2K) is the highest power term of “G(x)” in the formula. The relationship between ξ_m_ and δ_k_ is described by Eqs (11) and (12). The cumulative distribution function (CDF) of the extended probability density function may be formulated as:

$$F(x)=\int_{-\infty }^{x}\{\sum_{m=0}^{M}{\xi_{m}[G(\varepsilon )]}^{m}\}g(\varepsilon )d\varepsilon =\sum_{m=0}^{M}\left\{\frac{{\xi_{m}\cdot [G(x)]}^{m+1}}{m+1}\right\}.$$ (14)

### 3.3 Derivation of choice probabilities and likelihood function

Suppose there are “J” alternatives in the choice set and their random utility functions are U_1_, U_2_, …, U_J_. Let the utility U_j_ be expressed as the sum of the systematic component V_j_ and the random component ε_j_ (i.e., U_j_ = V_j_ + ε_j_). Assume that ε_j_ independently follows the extended distribution and its semi-nonparametric PDF and CDF are given as:

$$f_{j}(x)=\left\{\sum_{m_{j}=0}^{M_{j}}{\xi_{j,m_{j}}[G(x)]}^{m_{j}}\right\}g(x);$$ (15)

$$F_{j}(x)=\sum_{m_{j}=0}^{M_{j}}\left\{\frac{{\xi_{j,m_{j}}[G(x)]}^{m_{j}+1}}{m_{j}+1}\right\}.$$ (16)

The subscript “j” is added to allow ε_j_ in various random utilities to have different SNP distributions. In addition, three Lemmas, whose proofs are furnished in S1 Appendix, are used in the subsequent derivation of choice probabilities. Based on the utility maximization principle,

$$P(y=1)=P(U_{1}>U_{2\;}{,U}_{1}>U_{3\;}{,\ldots ,U}_{1}>U_{J\;})=P(V_{1}+\varepsilon_{1}>V_{2}+\varepsilon_{2},V_{1}+\varepsilon_{1}>V_{3}+\varepsilon_{3},\ldots ,V_{1}+\varepsilon_{1}>V_{J\;}+\varepsilon_{J}),$$

where “y” is a categorical choice variable indicating the specific alternative that is chosen. Then, P(y = 1) = P(ε_2_ < V_12_ + ε_1_,ε_3_ < V_13_ + ε_1_,…,ε_J_ < V_1J_ + ε_1_), where V_ij_ = V_i_ − V_j_.

$$\begin{array}{l} P\left(y=1\right)=\int_{-\infty }^{+\infty }F_{2}\left(V_{12}+\varepsilon_{1}\right)F_{3}\left(V_{13}+\varepsilon_{1}\right),\ldots ,F_{J}\left(V_{1J}+\varepsilon_{1}\right)f_{1}\left(\varepsilon_{1}\right)d\varepsilon_{1}\\ =\int_{-\infty }^{+\infty }\sum_{m_{2}=0}^{M_{2}}\left\{\frac{\xi_{2,m_{2}}{\left[G\left(V_{12}+\varepsilon_{1}\right)\right]}^{m_{2}+1}}{m_{2}+1}\right\}\ldots \sum_{m_{J}=0}^{M_{J}}\left\{\frac{\xi_{J,m_{J}}{\left[G\left(V_{1J}+\varepsilon_{1}\right)\right]}^{m_{J}+1}}{m_{J}+1}\right\}\left\{\sum_{m_{1}=0}^{M_{1}}\xi_{1,m_{1}}{\left[G\left(\varepsilon_{1}\right)\right]}^{m_{1}}\right\}g\left(\varepsilon_{1}\right)d\varepsilon_{1}. \end{array}$$

According to Lemma 1 in S1 Appendix, [G(ε)]^m^ = G[ε − ln(m)], where m > 0. Thus,

$$P(y=1)=\sum_{m_{1}=0}^{M_{1}}\sum_{m_{2}=0}^{M_{2}}\ldots \sum_{m_{J}=0}^{M_{J}}\frac{(m_{1}+1)\prod_{i=1}^{J}\xi_{i,m_{j}}}{\prod_{j=1}^{J}\left(m_{j}+1\right)}\int_{-\infty }^{+\infty }\{\prod_{j=2}^{J}G[V_{1j}+\varepsilon_{1}-\ln\left(m_{j}+1\right)]\}G[\varepsilon_{1}-\ln\left(m_{1}\right)]g(\varepsilon_{1})d\varepsilon_{1}.$$

Let the integral part in the formula be defined as "Int", i.e.,

$$Int=\int_{-\infty }^{+\infty }\{\prod_{j=2}^{J}G[V_{1j}+\varepsilon_{1}-\ln\left(m_{j}+1\right)]\}G[\varepsilon_{1}-\ln\left(m_{1}\right)]g(\varepsilon_{1})d\varepsilon_{1}.$$

According to Lemma 2 in S1 Appendix, $\left\{\prod_{j=2}^{J}G[V_{1j}+\varepsilon_{1}-\ln\left(m_{j}+1\right)]\right\}G[\varepsilon_{1}-\ln\left(m_{1}\right)]=G(\varepsilon_{1}+c)$,

where $c=-ln[e^{\ln\left(m_{2}+1\right)-V_{12}}+e^{\ln\left(m_{3}+1\right)-V_{13}}+\cdots e^{\ln\left(m_{J}+1\right)-V_{1J}}+e^{\ln\left(m_{1}\right)}]$. Then,

$Int=\int_{-\infty }^{+\infty }G(\varepsilon_{1}+c)g(\varepsilon_{1})d\varepsilon_{1}$. According to Lemma 3 in S1 Appendix,

$$\begin{array}{l} Int=\int_{-\infty }^{+\infty }G(\varepsilon_{1}+c)g(\varepsilon_{1})d\varepsilon_{1}=\frac{1}{1+e^{-c}}=\frac{1}{1+m_{1}+e^{\ln\left(m_{2}+1\right)-V_{12}}+e^{\ln\left(m_{3}+1\right)-V_{13}}+\cdots +e^{\ln\left(m_{J}+1\right)-V_{1J}}}=\\ \frac{e^{V_{1}}}{(1+m_{1})e^{V_{1}}+(1+m_{2})e^{V_{2}}+\cdots +(1+m_{J})e^{V_{J}}}=\frac{e^{V_{1}}}{\sum_{j=1}^{J}(1+m_{j})e^{V_{j}}}. \end{array}$$

By substituting "Int" into the choice probability expression, an elegant closed-form equation for the choice probability may be obtained:

$$P(y=1)=\sum_{m_{1}=0}^{M_{1}}\sum_{m_{2}=0}^{M_{2}}\ldots \sum_{m_{J}=0}^{M_{J}}\left\{\left[\frac{\prod_{i=1}^{J}\xi_{i,m_{j}}}{\prod_{j=1}^{J}\left(m_{j}+1\right)}]∙[\frac{(m_{1}+1)e^{V_{1}}}{\sum_{j=1}^{J}(m_{j}+1)e^{V_{j}}}\right]\right\}.$$ (17)

The derivation above is shown for the case when y = 1, but can be generalized to the situation where y = k. Without loss of generality,

$$P(y=k)=\sum_{m_{1}=0}^{M_{1}}\sum_{m_{2}=0}^{M_{2}}\ldots \sum_{m_{J}=0}^{M_{J}}\left\{\left[\frac{\prod_{i=1}^{J}\xi_{i,m_{j}}}{\prod_{j=1}^{J}\left(m_{j}+1\right)}]∙[\frac{(m_{k}+1)e^{V_{k}}}{\sum_{j=1}^{J}(m_{j}+1)e^{V_{j}}}\right]\right\}.$$ (18)

The log-likelihood function over the entire sample may be formulated as:

$$LL=\sum_{i=1}^{N}\sum_{k=1}^{J}{I(y}_{i}=k)\cdot ln[P(y_{i}=k)],$$ (19)

where I() is an indicator function; the subscript “i” is the index for an observed choice in the sample and “N” is the sample size. The log-likelihood function can be maximized to estimate model coefficients in the systematic component V_j_ as well as parameters in the vector δ_j_ that have been incorporated into $\xi_{i,m_{j}}$. When all M_j_ = 0, $P(y=k)=\frac{e^{V_{k}}}{\sum_{j=1}^{J}e^{V_{j}}}$ and the model reduces to the familiar MNL model. Thus, the proposed model may be considered a generalized multinomial logit model based on a semi-nonparametric approach.

## 4. Data and empirical estimation results

### 4.1 Data and modeling procedure

Data for the empirical study is extracted from the 2000 Swiss Microcensus travel survey. A sample consisting of 2,756 commuting trips reported by residents of Aargau Canton in Switzerland is used in this study to estimate models for commute mode choice. Four major commute modes are considered and defined as auto, transit, bicycle and walk. The sample market shares for these four alternatives show that the Aargau Canton of Switzerland depicts a multimodal transportation environment, where 57.62% of commuting trips are made by private auto and the remaining 42.38% of commuting trips are made by transit or non-motorized travel modes. In particular, the transit mode share is 15.86%, the bicycle mode share is 8.31%, and the walk mode share is 18.21%. The mode shares offer a sufficient number of observations in each travel mode, thus supporting the estimation of a mode choice model with multiple alternatives. In addition, multimodal network skim (level of service) data and commuters’ demographic and socioeconomic attributes are incorporated in the mode choice model specification.

The modeling effort started with the estimation of a simple MNL mode of mode choice. Model estimation results are presented in the first part of Table 2. Both level of service (LOS) attributes and commuters’ demographic and socioeconomic attributes are included as explanatory variables in the utility functions. Travel times, including auto in-vehicle time, transit in-vehicle time, and bicycle and walk times, exhibit significantly negative coefficients in the respective utility functions. Transit service frequency takes a significantly positive coefficient, indicating that a high service frequency would increase propensity of commuters to use transit. Model coefficients associated with demographic and socioeconomic attributes show that female commuters are less likely to use auto and bicycle modes. Low-income commuters are more likely to use transit or bicycle modes, while high-income commuters are less likely to use the transit mode. Commuters with lower education level are less likely to use auto than those with high education level. Older commuters are less likely to use public transit. All of the estimation results are behaviorally intuitive and consistent with expectations. The model’s log-likelihood value at convergence is -2495.646, corresponding to an adjusted likelihood ratio index of 0.1923 for the overall goodness-of-fit measure of the model.

**Table 2:** Model estimation results of MNL, SGMNL-11, SGMNL-21 and SGMNL-22.

Explanatory Variable	MNL		SGMNL-11		SGMNL-21		SGMNL-22	
	Est. Coef.	t-stat	Est. Coef.	t-stat	Est. Coef.	t-test	Est. Coef.	t-test
Auto Utility								
Constant	-0.0919	-1.03	0.9242	11.085	0.8312	10.113	0.8584	7.938
Auto in-vehicle time (min)	-0.0766	-11.41	-0.0698	-11.333	-0.0386	-10.217	-0.0455	-5.779
Commuter is female	-0.6618	-6.912	-0.5583	-6.568	-0.3915	-7.239	-0.4254	-6.307
Education level is less than or equal to middle school	-0.6461	-4.658	-0.4882	-4.226	-0.406	-5.02	-0.4319	-4.716
Transit Utility								
Constant	-2.373	-10.481	-2.1819	-10.167	-0.4047	-2.842	-1.3658	-8.807
Transit in-vehicle time (min)	-0.038	-5.915	-0.0311	-5.071	-0.0203	-5.202	-0.0235	-4.562
Transit service frequency per hour	0.0548	10.221	0.0531	10.288	0.033	10.444	0.0388	6.262
Commuter’s household monthly income is less than CHF 4,000	0.5536	2.432	0.4915	2.391	0.2537	2.224	0.2644	1.983
Commuter’s household monthly income is more than CHF 10,000	-0.3342	-2.243	-0.3181	-2.325	-0.1543	-2.094	-0.1836	-2.126
Commuter’s age (years)	-0.012	-2.818	-0.0119	-3.06	-0.0055	-2.603	-0.006	-2.394
Bicycle Utility								
Constant	-1.1107	-8.332	-1.0927	-8.227	-1.1433	-8.721	-1.1312	-8.539
Bicycle travel time (min)	-0.0756	-13.07	-0.0678	-11.569	-0.0571	-10.509	-0.0592	-10.172
Commuter is female	-0.4383	-2.805	-0.429	-2.768	-0.3041	-2.071	-0.3309	-2.17
Commuter’s household monthly income is less than CHF 4,000	0.7798	3.399	0.6945	3.223	0.696	3.276	0.6925	3.26
Walk Utility								
Walk travel time (min)	-0.0381	-24.515	-0.035	-22.002	-0.0312	-21.91	-0.0319	-20.304
Delta Values								
δ_1,1_	– –	– –	-1.1776	-1.699	-1.0236	-0.845	-0.9842	-0.778
δ_2,1_	– –	– –	– –	– –	-0.8471	-6.461	1.0613	1.625
δ_2,2_	– –	– –	– –	– –	– –	– –	-1.9138	-2.37
Model Statistics								
LL(β)	-2495.646		-2488.037		-2472.741		-2469.455	
χ^2^-test	– –		15.22		30.59		6.57	
Adj. ρ^2^(c)*	0.1923		0.1945		0.1991		0.1998	

* The log-likelihood value with constants only: LL(c) = -3104.836

Next, the proposed SGMNL (semi-nonparametric generalized multinomial logit) model is estimated to relax the standard Gumbel distribution for random components in modal utility functions. First, consider the specification in which K_j_ is set at 1, where “K” is the number of polynomials in Eq (7) and “j” is an index for travel mode (i.e., j = 1, 2, 3 or 4). When K_1_ = 1, it is found that the log-likelihood value improves from -2495.646 to -2488.037. As the current model nests the original MNL model, the likelihood ratio chi-square test may be applied to show that the improvement is statistically significant [i.e., (2495.646–2488.037) ×2 = 15.22 > 3.84, the critical chi-square value for one degree of freedom at a 95% confidence level]. This result strongly rejects the assumption of a standard Gumbel distribution for the random component in the auto utility function.

Model estimation results are presented in the second part of Table 2 and denoted as “SGMNL-11”. In this model, the signs of explanatory variable coefficients do not change from those obtained in the standard MNL model, but the magnitudes of coefficients in the auto utility function are found to differ. As expected, the alternative specific constant in the auto utility function changes substantially from -0.0919 to 0.9242 because the expectation of the new SNP distribution is very different from the expectation of the standard Gumbel distribution (Euler constant ≈ 0.577), and the alternative specific constant reflects this difference. An interesting finding is that the significance level of the single coefficient δ_1,1_ (as indicated by the t-statistic) is not as strong as that implied by the χ^2^ test for the overall model fit. However, it should be noted that the likelihood ratio test should be applied to determine whether a semi-nonparametric choice model form is more appropriate because the significance of multiple coefficients, and their contribution to overall goodness-of-fit, needs to be tested in most occasions.

After one significant coefficient δ_1,1_ is found for the first utility function, K_2_ in the second utility function is then set to 1 and δ_2,1_ is estimated. Estimation results for this model, denoted as “SGMNL-21”, are presented in the third part of Table 2. It can be seen that, after δ_2,1_ is introduced in the model specification, δ_1,1_ becomes insignificant but δ_2,1_ becomes highly significant as indicated by the t-statistics. The likelihood ratio test indicates that the model “SGMNL-21” with additional coefficient δ_2,1_ is significantly better than the model “SGMNL-11”, which does not include parameter δ_2,1_ [(2488.037–2472.741) × 2 ≈ 30.59 > 3.84]. The likelihood ratio test also shows that “SGMNL-21” is significantly better than the regular MNL model specification [(2495.646–2472.741) × 2 ≈ 45.81 > 5.99, the critical χ^2^ value corresponding to two degrees of freedom at a 95% confidence level]. Given that both “SGMNL-11” and “SGMNL-21” performed significantly better than the regular MNL model, both δ_1,1_ and δ_2,1_ should be retained in the SNP model. A comparison of coefficient estimates shows considerable differences across the “SGMNL-21”, “SGMNL-11”, and “MNL” models, particularly for the transit utility functions. This is consistent with the notion that the introduction of δ_1,1_ and δ_2,1_ will change the expectation and standard deviation of random components; both alternative specific constants and coefficients of explanatory variables change accordingly.

When δ_3,1_ or δ_4,1_ for bicycle and walk modes are introduced, no significant improvement is observed. In the interest of brevity, those estimation results are not presented here. The modeling effort now moves to the second stage, where the “K” value is increased to 2 and the coefficients δ_1,2_, δ_2,2_, δ_3,2_ and δ_4,2_ are introduced into the model one by one. In this stage, it is found that only the introduction of δ_2,2_ in the transit utility function significantly improves the overall model fit (χ^2^ test value = 6.57 > 3.84) while all other δ values do not. A final model estimation effort is performed, in which the “K” value is increased to 3 and parameter δ_2,3_ is introduced in the model. The maximum likelihood estimation procedure fails to converge, indicating that the sample of 2,756 choice observations may not be sufficient to support model estimation where the “K” value is increased to 3. Thus, the final best model is considered to be that which adopts a “K” value of 2 and introduces parameter δ_2,2_, in addition to parameters δ_1,1_ and δ_2,1_ introduced in “SGMNL-21”. This final model is designated “SGMNL-22”. If its model coefficients are compared with those in “SGMNL-21”, there is no substantial difference observed, except for the alternative specific constant and the coefficient associated with the “high-income” dummy variable in the transit utility function. As this is considered the final model, all subsequent comparisons are conducted between the MNL model and the final “SGMNL-22” model.

### 4.2 Plotting probability density distributions of random components in the *“SGMNL-22”* model

**Fig 3:**
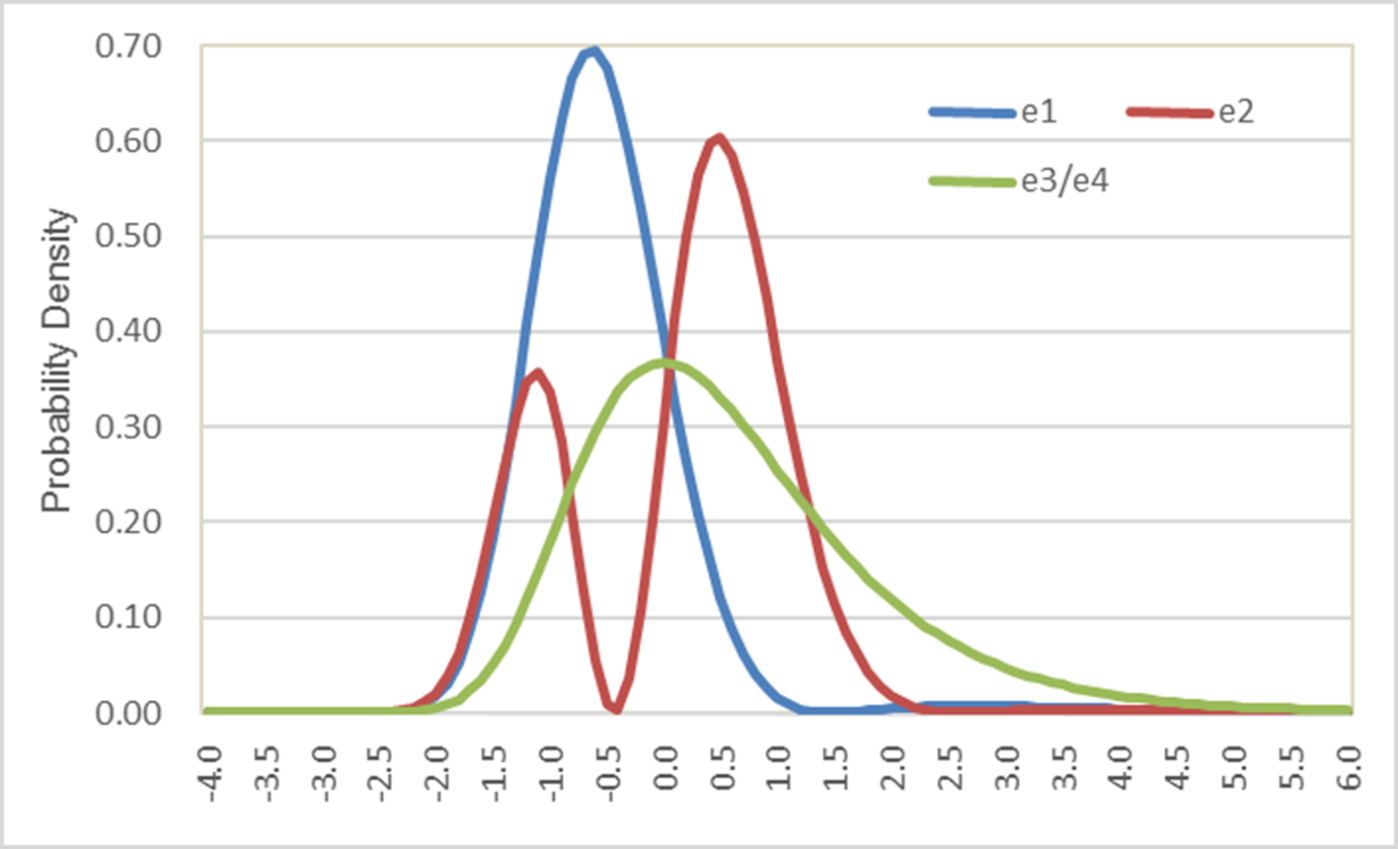
Probability density distributions of random components in the “SGMNL-22” model.

Fig 3 depicts the probability density distributions of random components in the “SGMNL-22” model. Eqs (11) and (12) are used to convert the estimated δ values to ξ values and then Eq (13) is used to compute the probability densities based on ξ values. The green curve represents the standard Gumbel distribution for random components in bicycle and walk mode utility functions (i.e., e3 and e4 in Fig 3). The blue curve represents the distribution of the random component in the auto utility function. The coefficient δ_1,1_ not only reduces the variance of the distribution of the random component but also shifts its mode towards the negative side by about 0.6 units. This helps explain why the alternative specific constant in the auto utility of the “SGMNL-22” model is substantially more positive than that in the MNL model. The positive alternative specific constant offsets the negative expectation of the new random component. The lower variance of the error distribution for the auto utility may be due to the existence of fewer unspecified or unobserved random factors associated with auto mode choice than with other mode choices. The distribution of the random component in the transit utility function (i.e., e2) presents an interesting pattern in the context of this study. With the inclusion of parameters δ_2,1_ and δ_2,2_ in the model (both of which are significant), “e2” depicts a bimodal distribution as shown by the red curve. The major mode on the right side is located near 0.6 and the minor one on the left side is near -1.2 on the coordinate axis. Based on this finding, it may be conjectured that there are two key groups of commuters mixed in the sample. One group of commuters has a positive attitude and inclination towards using transit and is associated with the major mode of the distribution. Meanwhile, a smaller group of commuters has a negative attitude towards transit and comprises the distribution near the minor mode. Although the exact source of the bimodal distribution is uncertain, the proposed SNP modeling method depicts the existence of such a phenomenon and exposes the potential limitation of using conventional MNL choice models that are based on unimodal distributional assumptions. Capturing the bimodal distribution in the choice model can help realize more consistent coefficient estimates and reliable policy sensitivities.

### 4.3 A comparison of aggregate marginal effects and elasticities

Coefficients in choice models usually do not directly reflect the impact of an explanatory variable on choice probabilities, particularly when the standard deviations of random components are scaled up or down, as in the transit or auto utility in the SGMNL model estimated in this study. To better understand differences in model sensitivity between MNL and SGMNL models, marginal effects and elasticities are computed and compared. In this subsection, aggregate marginal effects (*AME*) and aggregate elasticities (*AE*) with respect to level of service (LOS) variables are computed based on the following two equations:

$$AME=\frac{\partial (\sum_{i=1}^{N}P_{i}/N)}{\partial z_{i}}\approx \frac{\sum_{i=1}^{N}\left[P(x_{i},z_{i}+\Delta )-P(x_{i},z_{i})\right]}{N∙\Delta };$$ (20)

$$AE=\frac{\partial (\sum_{i=1}^{N}P_{i}/N)}{(\sum_{i=1}^{N}P_{i}/N)∙\partial z_{i}/z_{i}}\approx \frac{\sum_{i=1}^{N}\left[P(x_{i},z_{i}+\Delta ∙z_{i})-P(x_{i},z_{i})\right]}{\Delta ∙\sum_{i=1}^{N}P(x_{i},z_{i})}.$$ (21)

In the above equations, “P” represents the choice probability expression of the MNL or SGMNL model. “x_i_” represents a vector of explanatory variables except the one (i.e., z_i_) whose marginal effect or elasticity is being computed. “Δ” takes a value of 0.01 in this study as it is found that such a small interval provides sufficiently accurate estimates for “*AME*” and “*AE*” in both MNL and SGMNL models. Table 3 presents a comparison of computed “*AME*” and “*AE*” values between MNL and SGMNL-22 models. Relative differences in “*AME*” and “*AE*” are found to be considerable, which validates the notion that maximum likelihood estimators are inconsistent when distributional assumptions are violated. Such differences have important policy implications for transportation planning and management. For example, suppose a transportation authority intends to shift commuters from the auto mode to the transit mode by increasing transit service frequency. In predicting the number of commute drivers who will shift from auto to transit in response to the transit improvement, the conventional MNL model underestimates the elasticity with respect to transit service frequency by 25% (-0.082 vs -0.110).

**Table 3:** Comparisons of aggregate marginal effects (AME) and elasticities (AE).

Level-of-Service Variable	Auto	Transit	Bicycle	Walk
Aggregate Marginal Effects				
Model	SGMNL-22			
Auto in-vehicle time	-0.0140	0.0077	0.0021	0.0042
Transit in-vehicle time	0.0040	-0.0047	0.0002	0.0005
Transit service frequency per hour	-0.0066	0.0078	-0.0004	-0.0008
Bicycle travel time	0.0027	0.0006	-0.0044	0.0011
Walk travel time	0.0030	0.0006	0.0006	-0.0042
Model	MNL			
Auto in-vehicle time	-0.0154	0.0067	0.0030	0.0058
Transit in-vehicle time	0.0033	-0.0043	0.0004	0.0006
Transit service frequency per hour	-0.0048	0.0062	-0.0005	-0.0009
Bicycle travel time	0.0029	0.0007	-0.0055	0.0018
Walk travel time	0.0029	0.0006	0.0009	-0.0044
Aggregate Elasticities				
Model	SGMNL-22			
Auto in-vehicle time	-0.310	0.885	0.173	0.115
Transit in-vehicle time	0.126	-0.482	0.027	0.017
Transit service frequency per hour	-0.110	0.496	-0.072	-0.060
Bicycle travel time	0.077	0.080	-0.789	0.045
Walk travel time	0.164	0.163	0.160	-0.731
Model	MNL			
Auto in-vehicle time	-0.322	0.840	0.265	0.168
Transit in-vehicle time	0.111	-0.452	0.040	0.024
Transit service frequency per hour	-0.082	0.431	-0.091	-0.074
Bicycle travel time	0.089	0.096	-0.976	0.081
Walk travel time	0.169	0.173	0.266	-0.806

### 4.4 A comparison of disaggregate marginal effects and elasticities

The “*AME*” or “*AE*” presented in the previous subsection provide sample sensitivity to explanatory variables at the aggregate level and show how a level of service (LOS) variable, for example, affects market shares of alternatives based on the assumption that the sample is randomly drawn and can therefore represent the population shares well. However, aggregate measures of effects mask an important difference between MNL and SGMNL models. The MNL model has the IIA (Independence of Irrelevant Alternatives) property while the SGMNL model does not have this property. In order to illustrate this important difference between the two models, disaggregate marginal effects and elasticities are computed and compared for a specific individual commuter who is a 40 year old male with medium-level income and education level above middle school. The multimodal transportation level of service variables for this individual’s commute are as follows: auto in-vehicle time is 5 minutes; transit in-vehicle time is 8 minutes; transit service frequency is 6 times per hour; bicycle travel time is 12 minutes; and walk travel time is 35 minutes. Given these input variables for this specific commuter, both MNL and SGMNL-22 models are applied to compute choice probabilities of alternative travel modes. Results are shown in Table 4. There is a substantial difference in the choice probability of transit mode between the two models. The computations show that the MNL model returns a transit choice probability that is higher than that provided by the SGMNL-22 model by 41.8%, presumably because the model does not capture and reflect the bimodal distribution of the random component in the transit utility function.

**Table 4:** Comparisons of market shares and individual choice probabilities.

Statistics	Auto	Transit	Bicycle	Walk
Observed Sample Share	0.5762	0.1586	0.0831	0.1821
Predicted Sample Share from SGMNL-22	0.5738	0.1610	0.0829	0.1823
Predicted Sample Share from MNL	0.5762	0.1586	0.0831	0.1821
Predicted Individual Choice Probabilities from SGMNL-22 for Specific Commuter	0.5877	0.0388	0.1219	0.2516
Predicted Individual Choice Probabilities from MNL for Specific Commuter	0.5771	0.0550	0.1233	0.2446

Table 4 also presents a comparison of predicted means of market shares (i.e., $\sum_{i=1}^{N}{\hat{P}}_{i}/N$) over the entire sample. An appealing property of the MNL model is that it can replicate the observed sample shares perfectly using alternative specific constants in utility functions [1]. The SGMNL model does not have this feature, but the greatest difference occurs in the transit share where the relative difference is found to be only 1.5%, which is quite reasonable and acceptable.

The IIA property, which is a key feature of the MNL model, also manifests in the form of equal cross-elasticities [40]. Formulations similar to those expressed in Eqs (20) and (21) are applied to compute disaggregate marginal effects and elasticities with respect to LOS variables. The only difference is that the equations are applied to the specific individual commuter as opposed to all of the commuters in the sample. Results of the computations are presented in Table 5.

**Table 5:** Comparisons of disaggregate marginal effects and elasticities.

Level-of-Service Variable	Auto	Transit	Bicycle	Walk
Disaggregate Marginal Effects				
Model	SGMNL-22			
Auto in-vehicle time	-0.0145	0.0030	0.0038	0.0077
Transit in-vehicle time	0.0016	-0.0020	0.0002	0.0003
Transit service frequency per hour	-0.0026	0.0033	-0.0003	-0.0005
Bicycle travel time	0.0049	0.0004	-0.0066	0.0013
Walk travel time	0.0054	0.0004	0.0007	-0.0066
Model	MNL			
Auto in-vehicle time	-0.0187	0.0024	0.0055	0.0108
Transit in-vehicle time	0.0012	-0.0020	0.0003	0.0005
Transit service frequency per hour	-0.0017	0.0028	-0.0004	-0.0007
Bicycle travel time	0.0054	0.0005	-0.0082	0.0023
Walk travel time	0.0054	0.0005	0.0011	-0.0070
Disaggregate Elasticities				
Model	SGMNL-22			
Auto in-vehicle time	-0.124	0.390	0.154	0.154
Transit in-vehicle time	0.021	-0.417	0.010	0.010
Transit service frequency per hour	-0.026	0.519	-0.012	-0.012
Bicycle travel time	0.099	0.119	-0.646	0.063
Walk travel time	0.322	0.385	0.203	-0.910
Model	MNL			
Auto in-vehicle time	-0.162	0.221	0.221	0.221
Transit in-vehicle time	0.017	-0.287	0.017	0.017
Transit service frequency per hour	-0.018	0.311	-0.018	-0.018
Bicycle travel time	0.112	0.112	-0.793	0.112
Walk travel time	0.325	0.325	0.325	-1.004

It can be seen that cross-elasticities are equal in the MNL model, which reflects its IIA property. However, with unequal variances in auto and transit utilities in the SGMNL model, cross-elasticities for auto and transit choice probabilities are not equal, thus demonstrating that the SGMNL model does not possess the IIA property. However, because the random components in bicycle and walk utilities have equal variance, cross-elasticities for these two alternatives are still equal and therefore the IIA property holds for the bicycle and walk modes even in the case of the SGMNL model. This is similar to the situation where two alternatives belong to the same nest in a nested logit model.

### 4.5 A comparison of changes in transit choice probability in response to a service frequency improvement

**Fig 4:**
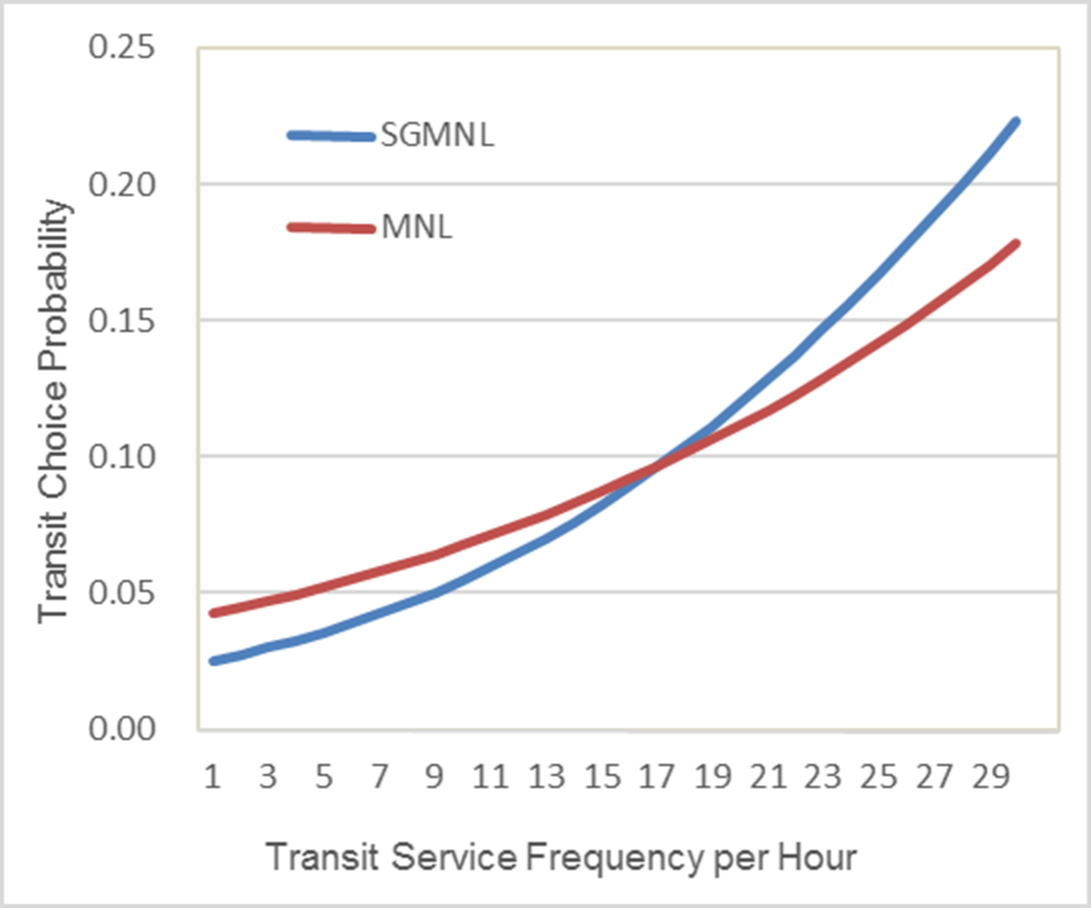
Transit choice probability for a specific commuter in response to an improvement in service frequency.

To further illustrate the policy implications of alternative model forms, changes in transit choice probability predicted by the two models in response to a service frequency improvement are compared for the specific individual commuter considered previously. The result of this comparison is presented in Fig 4. Relative to the SGMNL model, the MNL model overestimates the transit choice probability when the service frequency is low (<18 per hour) but underestimates it when the service frequency is high (≥18 per hour). A service frequency of 18 transit vehicles per hour is quite high, reflecting a headway of just over three minutes. Given that most real-world transit services operate at frequencies less than 18 vehicles per hour, it appears that the MNL model is likely to overestimate the transit choice probability relative to the SGMNL model. In this particular example, when the service frequency is very low (≤4 per hour), the relative difference between the predicted transit choice probabilities computed from the MNL and SGMNL models can exceed 50%.

## 5. Conclusions

In this paper, a semi-nonparametric generalized multinomial logit (SGMNL) model is formulated and developed by applying orthonormal Legendre polynomials to extend the standard Gumbel distribution that lies at the core of multinomial logit models applied in practice. The semi-nonparametric function with flexible forms can represent a probability density function for a large family of multimodal distributions. Unlike the existing semi-nonparametric modeling method which is applied to binary choice situations in the econometric literature, the proposed method allows for modeling multinomial choices, which are typically encountered in travel-related choice behavior analysis and travel demand modeling. The advantage of the proposed method is that the formulation results in a closed-form likelihood function and standard maximum likelihood estimation methods can be applied for parameter estimation. Thus, the model estimation procedure is computationally efficient and free from simulation-based complexity or errors.

The proposed modeling method is applied to an empirical setting of commute travel mode choice among four alternatives (auto, transit, bicycle and walk), based on travel survey and network skim (level of service) data from the Canton of Argau in Switzerland. It is found that the distribution of the random component in the auto utility function is similar to a Gumbel distribution, but has substantially smaller variance. More notably, the random component in the transit utility function follows a bimodal distribution, which indicates a significant departure from and violation of the assumption of a Gumbel distribution. Unequal variances accommodated in the formulation allow the semi-nonparametric model to be free of the limitations of the IIA property that are inherent to the multinomial logit model. The semi-nonparametric model specifications are found to offer superior goodness-of-fit when compared with the MNL model. The violation of the standard Gumbel distribution assumption in the multinomial logit model leads to inconsistent coefficient estimates, marginal effects, elasticities and choice probabilities. In the empirical context considered in this study, the multinomial logit model is found to overestimate the predicted transit choice probability relative to the semi-nonparametric model for transit service scenarios commonly encountered in the real world.

A few limitations of the proposed method and directions for future research are worthy of note. First, it may be challenging to directly apply the proposed method to model choice behaviors in the context of a large choice set (e.g. [41]). The likelihood function, depicted in Eq (18), involves multiple levels of summations and the number of levels is dependent on the number of alternatives in the choice set. Thus, the computational complexity will increase geometrically with an increase in the number of alternatives in the choice set. Future research should focus on reducing computational complexity in the context of large choice sets. Second, the proposed model is developed based on the assumption that random components in utility functions are mutually independent. However, this assumption may not hold in empirical settings. In future research, there may be the potential to introduce correlations in joint semi-nonparametric distributions and develop nested or cross-nested versions of the proposed semi-nonparametric multinomial choice model. Third, it is uncertain whether the empirical results of this study, in which the random component of the transit utility is found to follow a bimodal distribution, are valid in different geographical and modal contexts. Conducting studies similar to this one in different contexts would help shed light on the generalizability of results reported in this paper.

## Supporting information

S1 Appendix Appendix. (DOCX)

S1 Data Data. (CSV)

S1 Fig Comparisons of semi-nonparametric probability densities when K = 1. (TIF)

S2 Fig Comparisons of semi-nonparametric probability densities when K = 2. (TIF)

S3 Fig Probability density distributions of random components in the “SGMNL-22” model. (TIF)

S4 Fig Transit choice probability for a specific commuter in response to an improvement in service frequency. (TIF)

S1 Table An example of “c” matrix. (TIF)

S2 Table Model estimation results of MNL, SGMNL-11, SGMNL-21 and SGMNL-22. (TIF)

S3 Table Comparisons of aggregate marginal effects (AME) and elasticities (AE). (TIF)

S4 Table Comparisons of market shares and individual choice probabilities. (TIF)

S5 Table Comparisons of disaggregate marginal effects and elasticities. (TIF)
