# Supplementary material for: On the development of a semi-nonparametric generalized multinomial logit model for travel-related choices
Source: PLoS One. 2017 Oct 26;12(10):e0186689. doi: 10.1371/journal.pone.0186689 (PMC5658062; doi:10.1371/journal.pone.0186689)
Supplement: S1 Appendix — (DOCX) [file pone.0186689.s001.docx]

# Appendix

Lemma 1: $\left[ G\left( \varepsilon\right) \right]^{m}=G[\varepsilon-ln(m)]$, where *m* > 0.

Proof: $\left[ G\left( \varepsilon\right) \right]^{m}={exp(-e^{-\varepsilon})}^{m}=exp\left( -m\cdot e^{-\varepsilon} \right)=exp\left\{ -e^{-\left[ \varepsilon-ln(m) \right]} \right\}=G[\varepsilon-ln(m)]$.

Lemma 2: $G\left( a+\varepsilon\right)G\left( b+\varepsilon\right)=G\left( c+\varepsilon\right),$ where $c=-ln( e^{-a}+e^{-b})$. More generally, $\prod_{j=1}^{J} G\left( a_{j}+\varepsilon\right)=G\left( c+\varepsilon\right),$ where $c=-ln\left( \sum_{j=1}^{J} e^{-a_{j}} \right)$.

Proof:$G\left( a+\varepsilon\right)G\left( b+\varepsilon\right)=exp\left( -e^{-a-\varepsilon} \right)\exp\left( -e^{-b-\varepsilon} \right)=exp(-e^{-a-\varepsilon}-e^{-b-\varepsilon})$

$=\exp\left[ -e^{-\varepsilon}\left( e^{-a}+e^{-b} \right) \right]=exp \left[ -e^{-\varepsilon+ln\left( e^{-a}+e^{-b} \right)} \right]=G\left( c+\varepsilon\right)$, where $c=-ln( e^{-a}+e^{-b})$.

More generally, $\prod_{j=1}^{J} G\left( a_{j}+\varepsilon\right)=\prod_{j=1}^{J} \exp\left( -e^{-a_{j}-\varepsilon} \right)=exp(\sum_{j=1}^{J} -e^{-a_{j}-\varepsilon})$

$=\exp\left[ -e^{-\varepsilon}\sum_{j=1}^{J} e^{-a_{j}} \right]=exp \left[ -e^{-\varepsilon+ln\left( \sum_{j=1}^{J} e^{-a_{j}} \right)} \right]=G(c+\varepsilon)$, where $c=-ln\left( \sum_{j=1}^{J} e^{-a_{j}} \right)$.

Lemma 3: $\int_{-}^{+} G\left( x+c \right)g\left( x \right)dx=\frac{1}{1+e^{-c}}$.

Proof: $\int_{-}^{+} G\left( x+c \right)g\left( x \right)dx=\int_{-}^{+} \exp\left( -e^{-x-c} \right)\exp\left( -e^{-x} \right)e^{-x}dx$

$=-\int_{-}^{+} \exp\left( -{e^{-c}e}^{-x} \right)\exp\left( -e^{-x} \right)d\left( e^{-x} \right)$.

Let $y=e^{-x}$ , then $\int_{-}^{+} G\left( x+c \right)g\left( x \right)dx=-\int_{+}^{0} \exp\left( -y\cdot e^{-c} \right)\exp\left( -y \right)d\left( y \right)$

$=-\int_{+}^{0} \exp\left[ -y\cdot{(1+e}^{-c}) \right]d\left( y \right)$.

Let $k={(1+e}^{-c})$, then $\int_{-}^{+} G\left( x+c \right)g\left( x \right)dx=-\int_{+}^{0} e^{-ky}d\left( y \right)=\frac{1}{k}\int_{+}^{0} e^{-ky}d\left( -ky \right)$.

Let $q=-ky$, then $\int_{-}^{+} G\left( x+c \right)g\left( x \right)dx=\frac{1}{k}\int_{-}^{0} e^{q}dq =\frac{1}{k}\left[ e^{q} \right]_{-}^{0}=\frac{1}{k}=\frac{1}{1+e^{-c}}$.
