# Supplementary figures and images for: On the development of a semi-nonparametric generalized multinomial logit model for travel-related choices
Source: PLoS One. 2017 Oct 26;12(10):e0186689. doi: 10.1371/journal.pone.0186689 (PMC5658062; doi:10.1371/journal.pone.0186689)

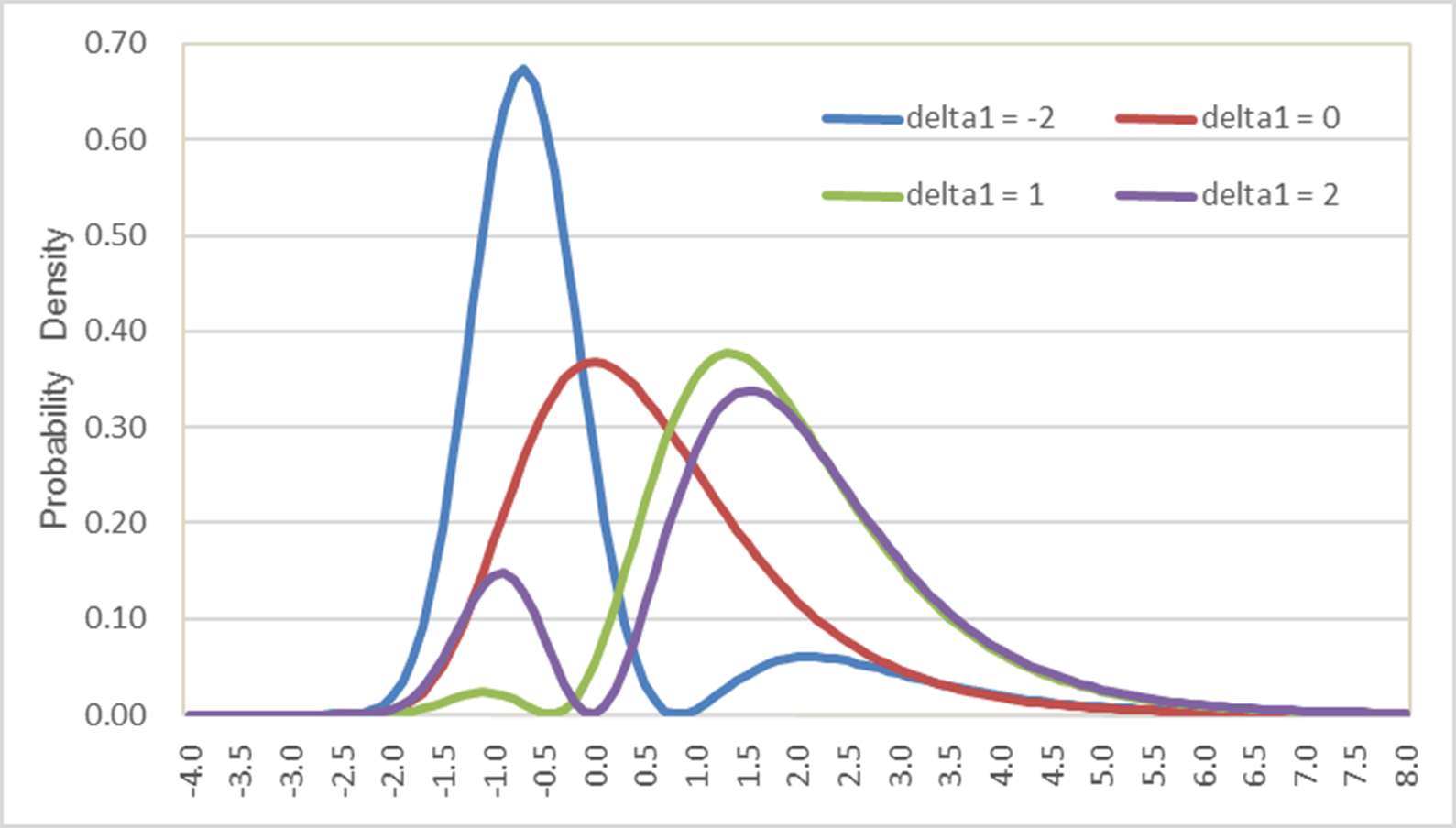

Supplement: S1 Fig — (TIF) [file pone.0186689.s003.tif]

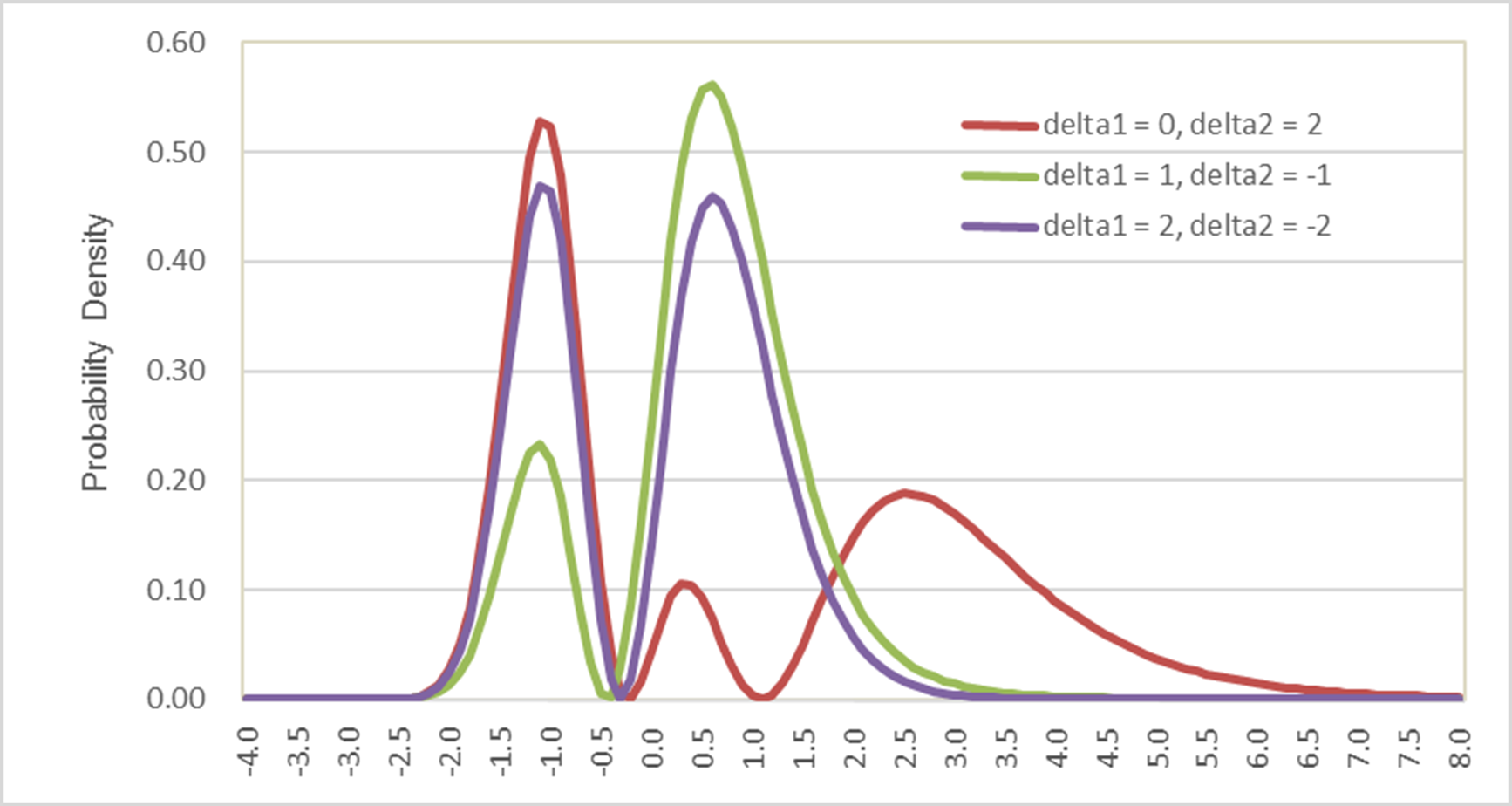

Supplement: S2 Fig — (TIF) [file pone.0186689.s004.tif]

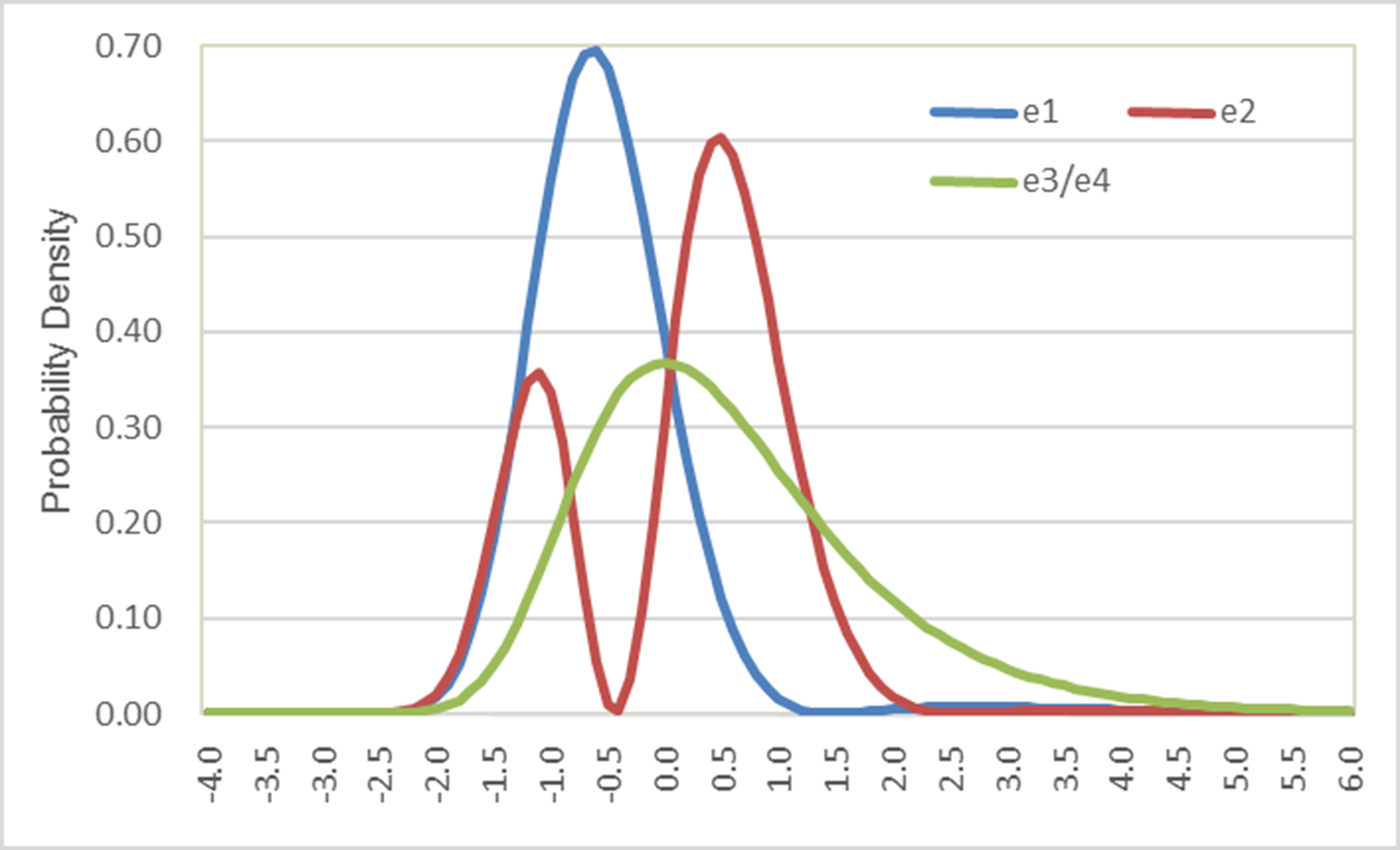

Supplement: S3 Fig — (TIF) [file pone.0186689.s005.tif]

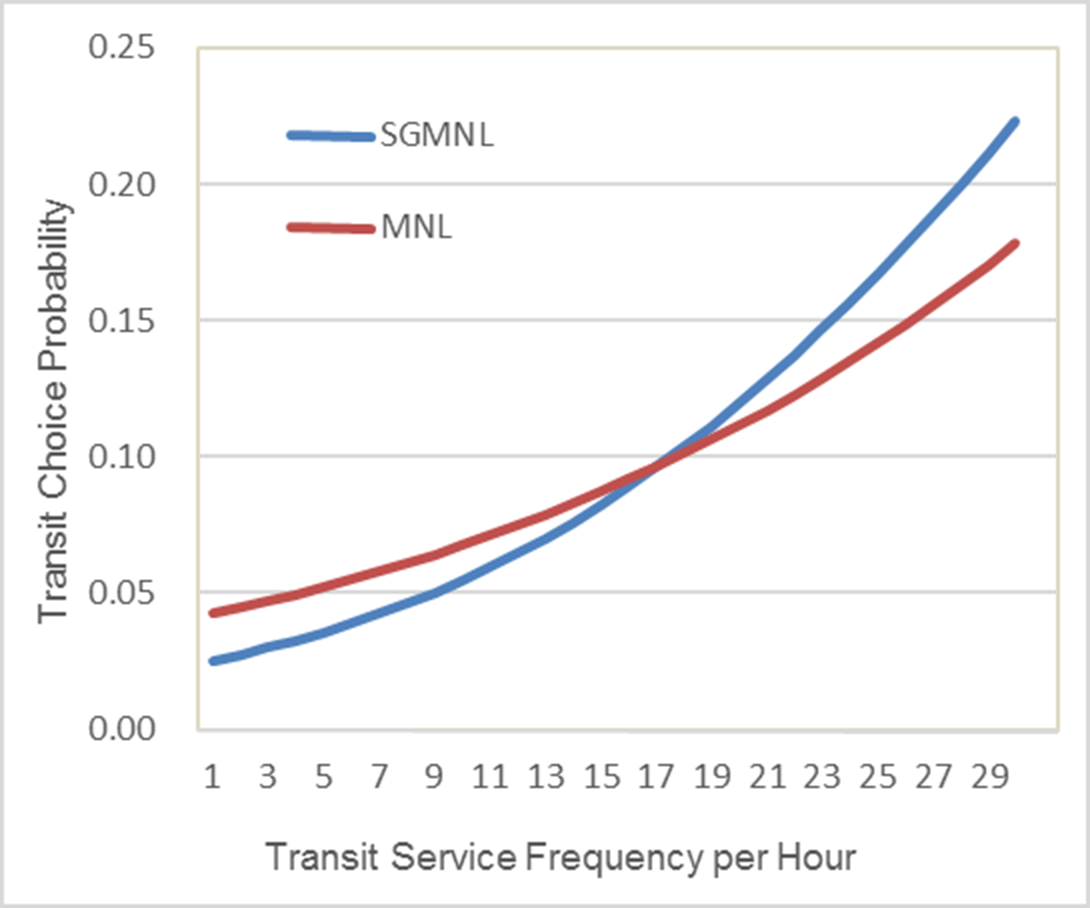

Supplement: S4 Fig — (TIF) [file pone.0186689.s006.tif]

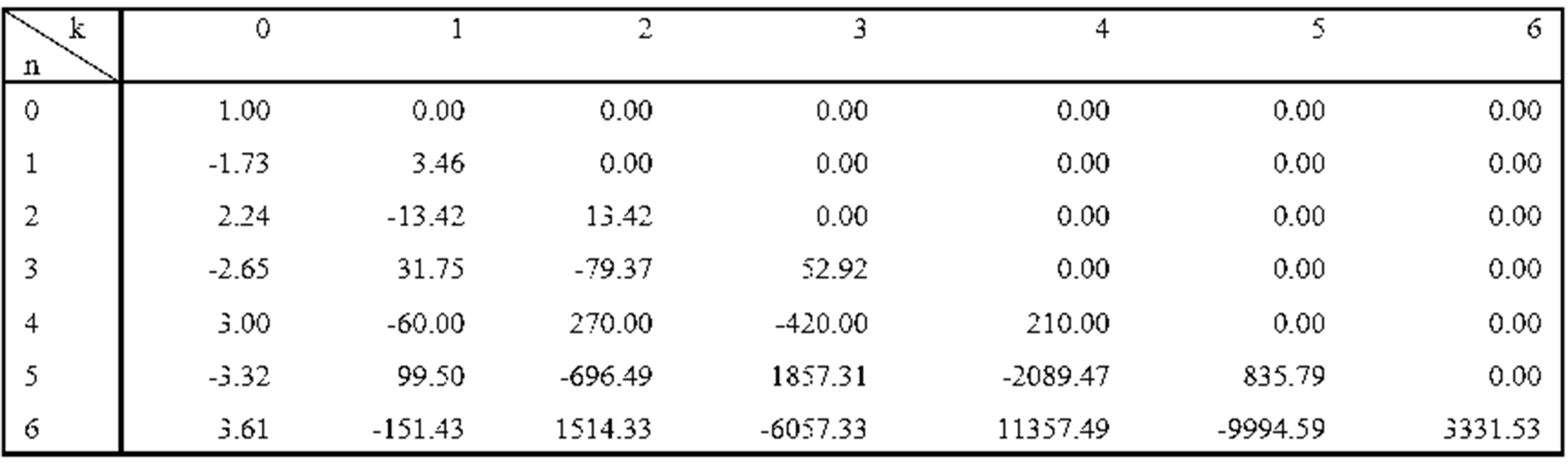

Supplement: S1 Table — (TIF) [file pone.0186689.s007.tif]

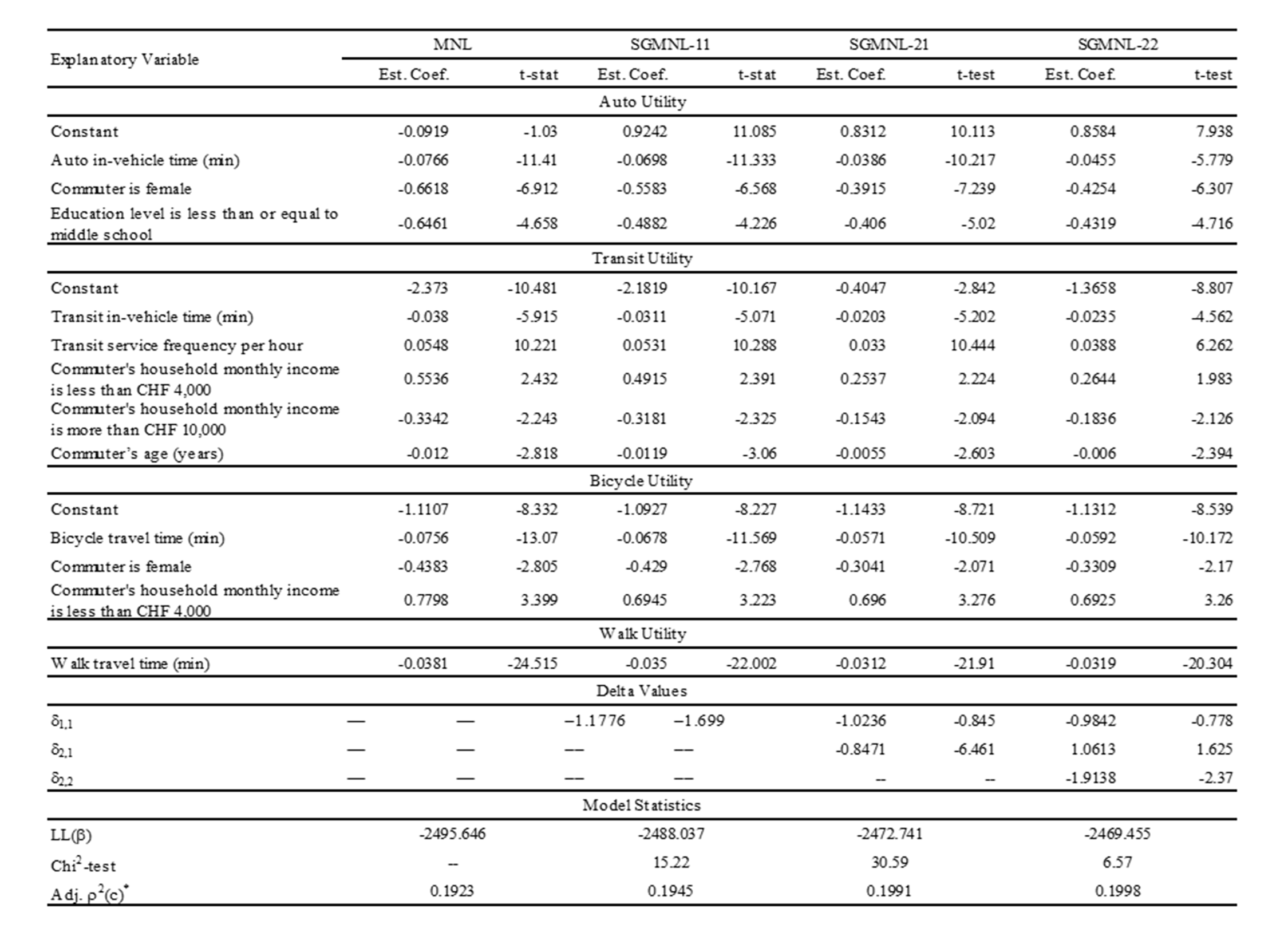

Supplement: S2 Table — (TIF) [file pone.0186689.s008.tif]

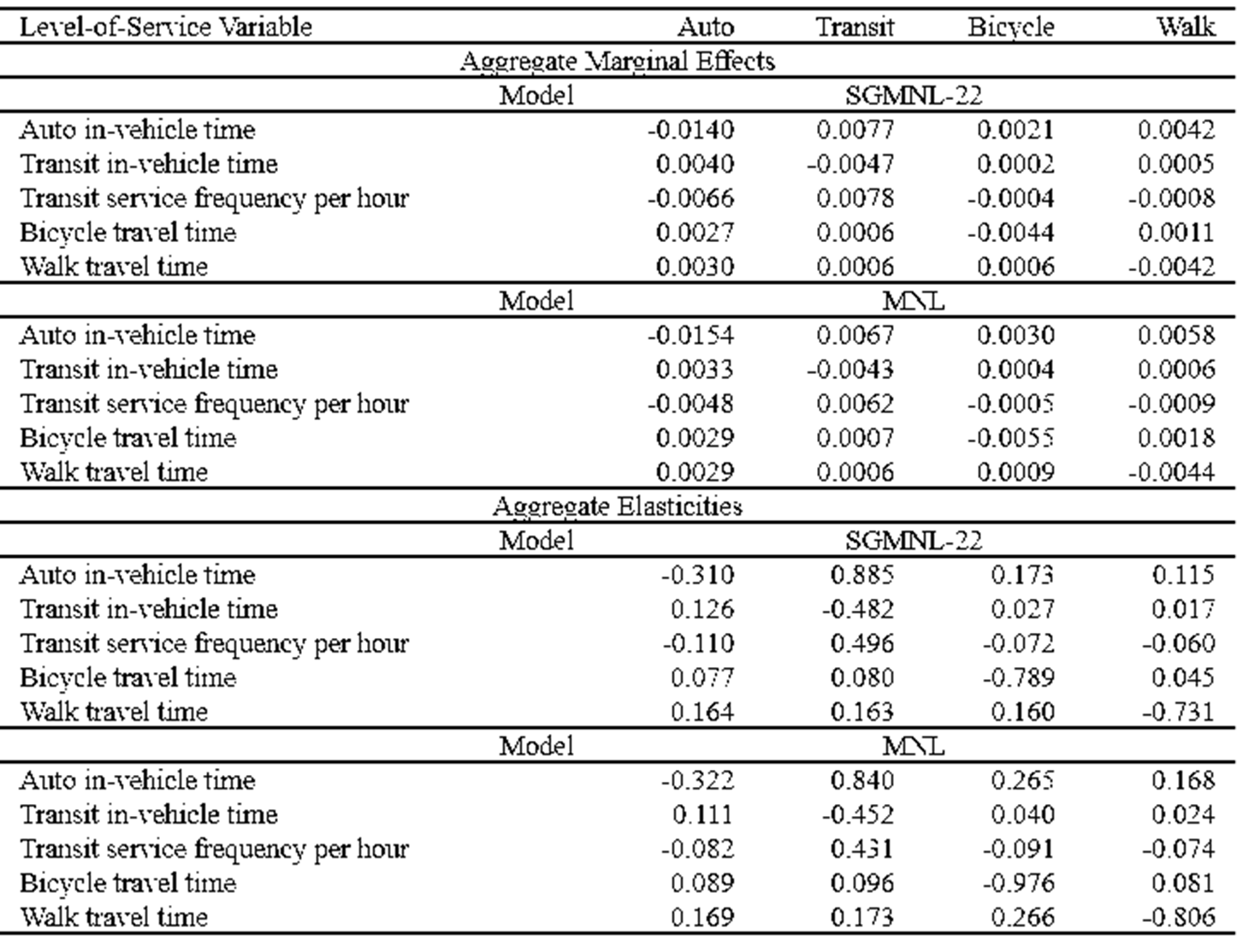

Supplement: S3 Table — (TIF) [file pone.0186689.s009.tif]

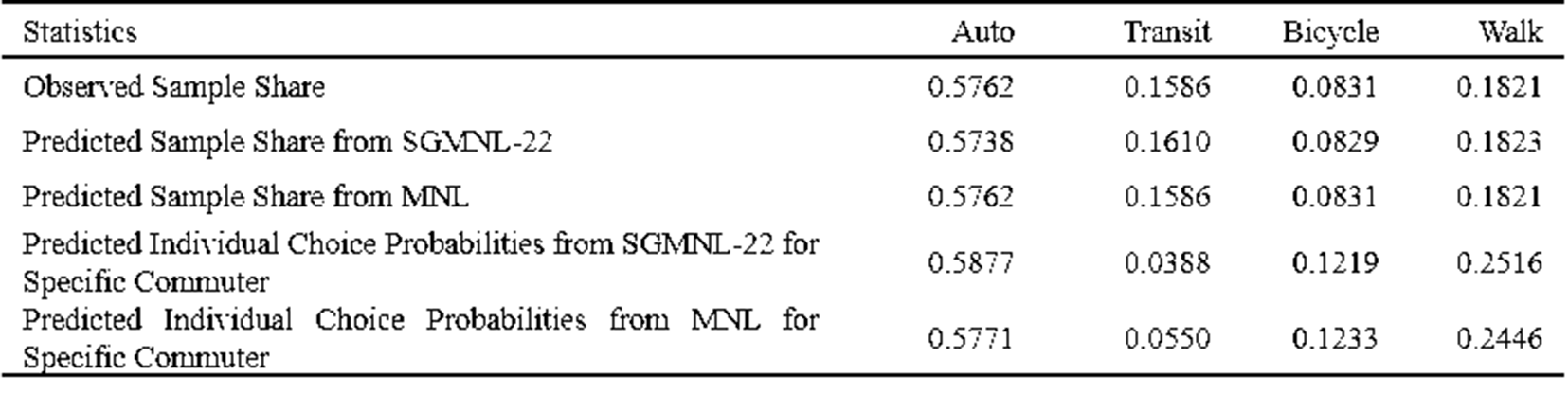

Supplement: S4 Table — (TIF) [file pone.0186689.s010.tif]

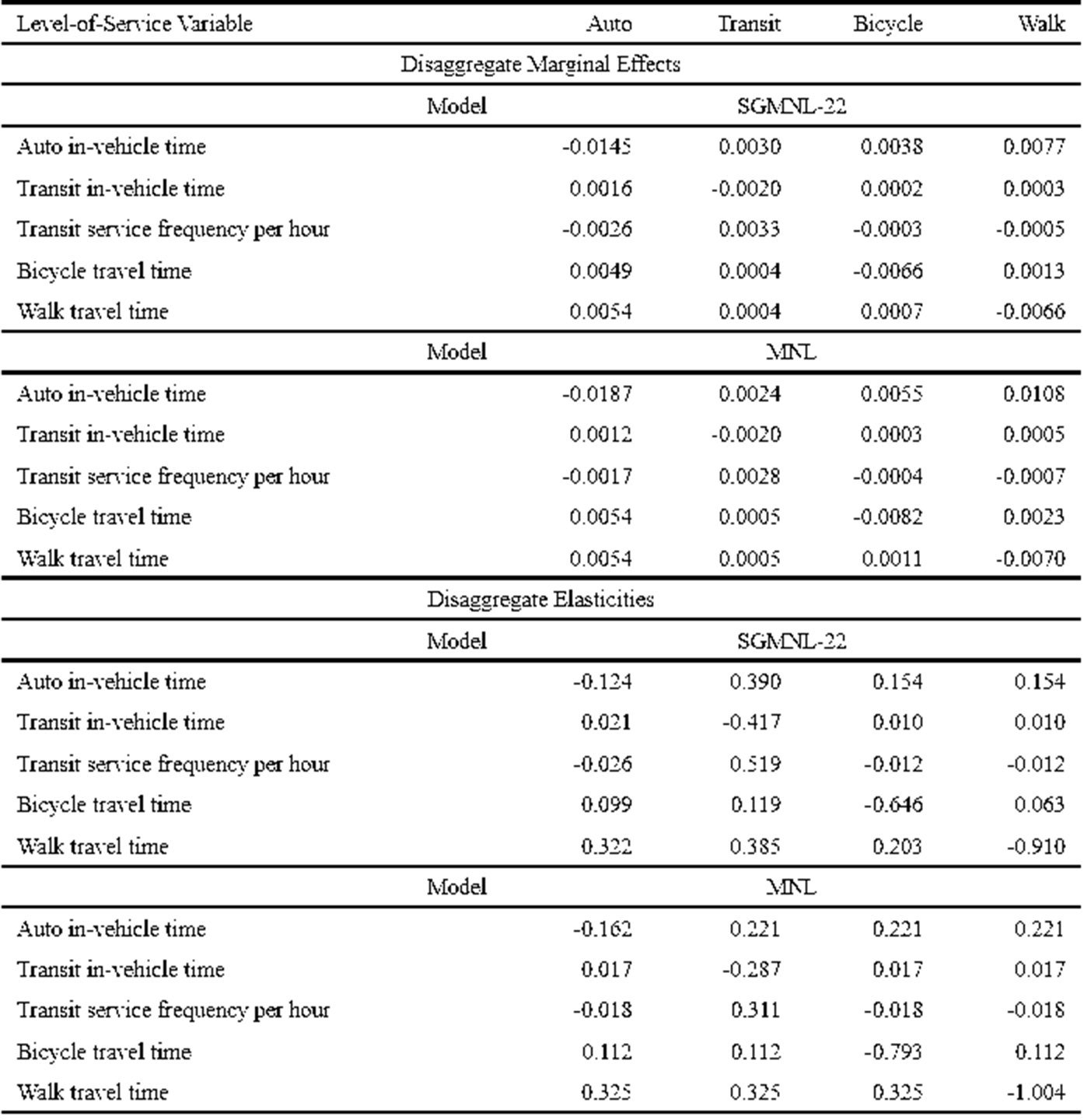

Supplement: S5 Table — (TIF) [file pone.0186689.s011.tif]
